# Noninvasive *in vivo* human multiphoton microscopy: a key method in proving nanoparticulate zinc oxide sunscreen safety

**DOI:** 10.1117/1.JBO.25.1.014509

**Published:** 2020-01-14

**Authors:** Yousuf H. Mohammed, Deborah S. Barkauskas, Amy Holmes, Jeffrey Grice, Michael S. Roberts

**Affiliations:** a University of Queensland, University of Queensland Diamantina Institute, Therapeutics Research Group, Woolloongabba, Queensland, Australia; b University of South Australia, Basil Hetzel Institute for Translational Medical Research, The Queen Elizabeth Hospital, School of Pharmacy and Medical Sciences, Adelaide, Australia

**Keywords:** optics, multiphoton, tomography, microscopy, femtosecond laser, zinc oxide nanoparticle, sunscreen, spectral imaging, FLIM, phosphorescence lifetime imaging, second harmonic generation, product safety

## Abstract

We describe the contribution of our *in vivo* multiphoton microscopy (MPM) studies over the last ten years with DermaInspect^®^ (JenLab, Germany), a CE-certified medical tomograph based on detection of fluorescent biomolecules, to the assessment of possible penetration of nanoparticulate zinc oxide in sunscreen through human skin. At the time we started our work, there was a strong movement for the precautionary principle to be applied to the use of nanoparticles in consumer products due to a lack of knowledge. The combined application of different MPM modalities, including spectral imaging, fluorescence lifetime imaging, second harmonic fluorescence generation, and phosphorescence microscopy, has provided overwhelming evidence that nanoparticle zinc oxide particles do not penetrate human skin when applied to various skin types with a range of methods of topical sunscreen application. MPM has also been used to study the viable epidermal morphology and redox state in supporting the safe use of topical zinc oxide nanoparticles. The impact of this work is emphasized by the recent proposed rule by the United States FDA on Sunscreen Drug Products for Over-the-Counter Human Use, which listed only zinc oxide and titanium dioxide of the currently marketed products to be generally recognized as safe and effective.

## 1 Introduction

Researchers at the forefront of advances in technology have always had the flow-on benefits of being able to apply that technology in addressing previously unresolvable key concerns in biology and other disciplines. These findings may, in turn, stimulate the next wave of technological advances or refinements, resulting in further research and development in resolving particular ill-defined issues of interest. One such example of a vital juxtaposition is the role played by the commercial development of multiphoton microscopy (MPM) for human studies in the assessment and, ultimately, the regulatory approval of the nano-sized and transparent zinc oxide sunscreens, which we present here as a case study. We emphasize that this is only one of many examples of how this commercial MPM has been used. Noninvasively obtaining optical images at various depths of intact skin on humans using MPM is known as multiphoton tomography (MPT). König et al. have pointed out that unique tomographs for more than 2000 patients and volunteers have been obtained in Europe, Australia, and Asia and that the next wave of technology has begun with femtosecond laser nanoprocessing microscopes now being employed for targeted delivery and deposition in body organs, optical transfection, optical cleaning of stem cells, tracking of microRNAs, and imaging of human corneas.^1^

The significance of the findings reported in this case study is emphasized by the role played by sunscreens in preventing photo-aged skin, sunburn, and the various sun-induced skin cancers. In the United States alone, about 9500 people are diagnosed with skin cancer every day, and it is estimated that nonmelanoma skin cancer, including basal cell carcinoma and squamous cell carcinoma, affects more than 3 million Americans a year.^2^ These have had an average annual treatment cost of $8.1 billion for the ∼4.9 million US adults affected. As the most preventable risk factor for all skin cancers is exposure to UV light, the American Academy of Dermatology encourages individuals to use a broad-spectrum, water-resistant sunscreen with a sun protection factor (SPF) of 30 or higher to protect their skin from the sun’s harmful UV rays.

Of the available sunscreens, only zinc oxide provides effective protection across the UV band range of 240 to 400 nm, covering UVC (240 to 280 nm), UVB (280 to 320 nm), UVA 2 (320 to 340 nm), and UVA 1 (340 to 400 nm).^3^ It is therefore of special interest to optimize and improve its action as a sunscreen suitable for everyday use. The use of any consumer product raises the question as to whether it is safe; as a result, there have been careful and extensive evaluations by regulatory authorities to prove safety. For instance, the European Union Commission that regulates cosmetics evaluated topical zinc oxide safety and concluded in 2003 that “in general, zinc oxide may be considered as a nontoxic substance, including when used in cosmetic products” but there was some concern in “the risk assessment of micronized (∼0.2  μm) zinc oxide, which may be coated by other compounds, and which is used as an ingredient in sunscreen formulations” and for which “findings need to be clarified by appropriate investigations *in vivo*,” noting that “there is a lack of reliable data on the percutaneous absorption of micronized zinc oxide.”^4^ Not long after this, concerns were raised about the safety of nanoparticles, including nanoparticle-based sunscreens, which had the advantage over existing opaque inorganic sunscreens of also being transparent.^5^

**Fig. 1:**
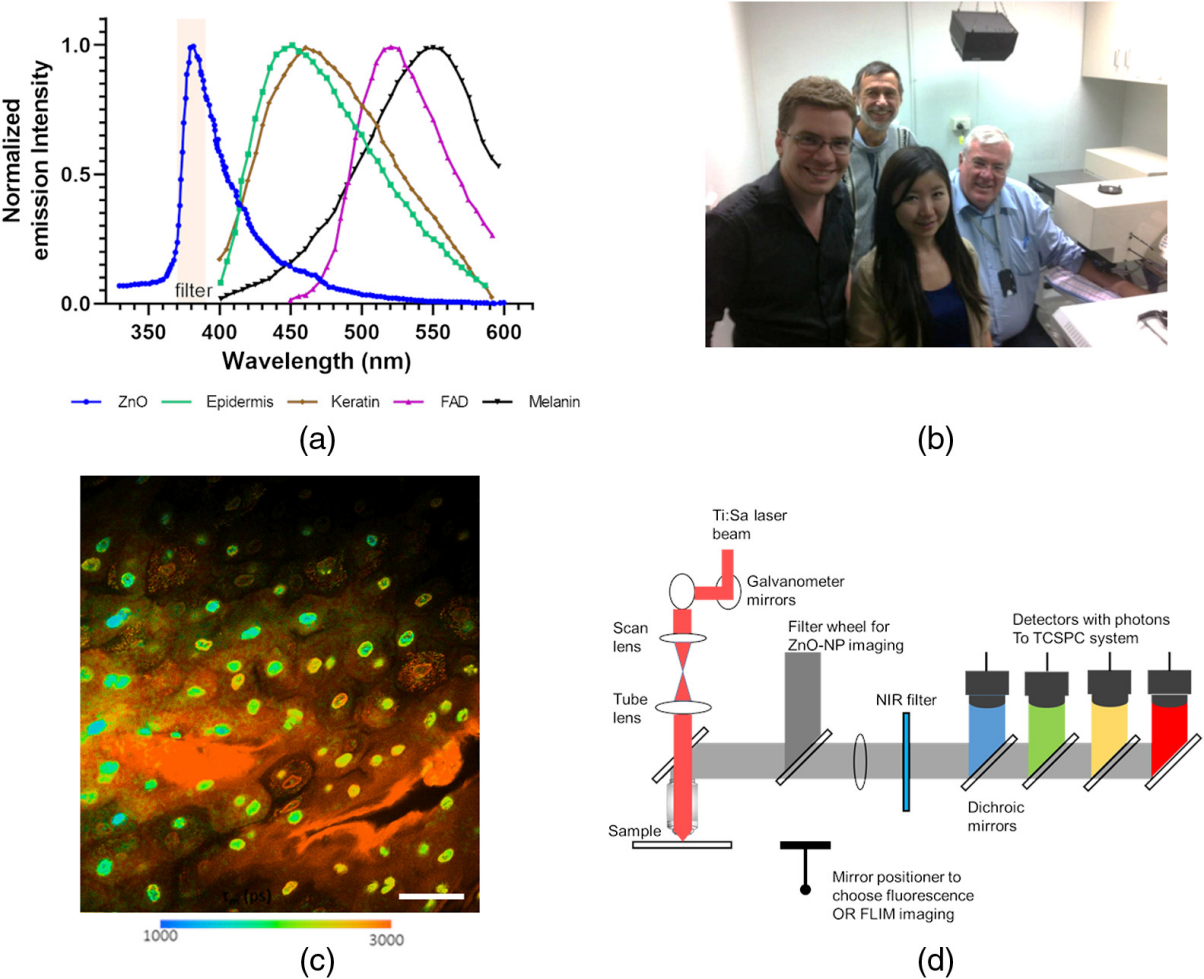
Essentials of MPM-spectral-FLIM skin imaging. (a) Emission spectra for main endogenous fluorophores in the skin and for nano zinc oxide. (Redrawn with permission from Ref. 8.) (b) Skin imaging using the DermaInspect^®^ system in the Therapeutics Research Center at University of Queensland (UQ). (c) Typical high-resolution image of viable epidermal cells after acriflavine treatment with the DermaInspect^®^, color-coded to visualize mean fluorescence lifetime, $\tau_{m}$. Scale bar is 40  μm. (d) Schematic of detectors in the DermaInspect^®^ based at UQ.

We became involved in MPM imaging of zinc oxide on human skin as a result of our initial *in vitro* studies on nano zinc oxide skin penetration through human skin^6^ and began using MPM to study nanoparticulate zinc oxide penetration through human skin *in vivo*. We first presented our initial findings at an FDA public forum in 2006.^7^ Here, we document the MPM studies that we and others have undertaken over the last ten years with the *in vivo* CE-certified medical tomograph DermaInspect^®^ system [see Fig. 1(b)], developed by Karsten König of JenLab, to show that zinc oxide nanoparticles (ZnO NPs) do not penetrate human skin. We describe the imaging of ZnO NPs via different methods:^9^^–^^14^ (1) fluorescence imaging using spectral filters, (2) fluorescence lifetime imaging microscopy (FLIM) analysis of fluorescence lifetimes $\tau_{1}$ and $\tau_{2}$, (3) second harmonic generation (SHG) and hyper-Rayleigh scatter (HRS), (4) multiphoton excited photoluminescence (MEP) defined by the FLIM fluorescence lifetime amplitude $a_{1}\%$, and (5) photoluminescence offset. We also show through stained and MPM-imaged cyrosections of human skin that zinc ions, generated on the breakdown of ZnO NPs, penetrate into viable skin. Further, we describe our use of MPM to assess possible morphological changes in the morphology and redox state of the viable epidermis in the assessment of the possible direct viable epidermal toxicity of nanoparticulate zinc oxide.

## 2 Noninvasively Imaging Human Skin with MPM

MPM has proven to be the method of choice for human skin imaging. A key limitation in using conventional fluorescence and confocal microscopy for *in vivo* imaging of tissues is the potential UVA-induced photodamage for near-infrared radiation wavelengths below 650 nm. Much less damage may be anticipated with MPM as the out-of-focus region will generally be unaffected by low energy photons from low power, femtosecond (fs) laser pulses at wavelengths greater than 650 nm. While there remains a possibility of adverse photodamage in the tiny focal volume where the nonlinear absorption occurs, Fischer et al.^15^ suggested that the risk of damage for femtosecond infrared radiation in human skin imaging is negligible and not increased by multiple imaging. They recommend the avoidance of multiple imaging of the identical area within a 3- to 6-month period and a limit of 10 exposures to the same area in a lifetime to keep the local tumor risk to a minimum. UVA radiation under imaging conditions is much less than 0.5% and an order of magnitude less than the natural exposure risk of 3% to 10%, for instance, in the neck area.

An additional advantage in imaging at >650-nm near-infrared radiation wavelengths is there is an increased depth of tissue penetration of up to about 1350 nm where water strongly absorbs light and attenuates the radiation. Consequently, there is an effective near-infrared window in biological tissue (also known as the optical window) from 650 to 1350 nm. This window is also associated with the greatest depth of light penetration in tissue, low one-photon absorption, and low scattering coefficients for unstained cells and tissues. A range of endogenous skin fluorophores (NADH, melanin, keratin, flavin, and collagen) show either two photon fluorescence or SHG, enabling imaging without the use of external staining agents [Fig. 1(a)]. Studies by König and others have suggested that multiphoton biopsies provide the highest resolution of all *in vivo* tissue imaging techniques. The MPM has a high signal depth, enables optical sectioning within seconds, and has a high sensitivity as a result of pinhole-free single photon counting, with a <0.5-μm lateral resolution and 1- to 2-μm axial resolution, giving high quality noninvasively acquired viable epidermal images^16^ [Fig. 1(c)].

The range of endogenous fluorophores in human skin provides both an opportunity and challenge in the imaging of exogenously applied solutes. The advantage is that they allow the detailed morphological structure of the various skin layers to be seen by noninvasive multiphoton imaging. Table 1 shows that various endogenous solutes in the skin have varying excitation and emission wavelengths. In principle, both could be modified to correspond to the maximum absorbance and emission for a given compound to give a selective measurement of that compound relative to others. In ophthalmic imaging, it is common to vary the excitation wavelength ($\lambda_{\mathrm{ex}}$) of fluorescein, to separate the fluorescein from its glucuronide as both have a similar emission wavelength ($\lambda_{em}$) but have differing single-photon excitation wavelengths (fluorescein $\lambda_{\mathrm{ex}}$ 457.9 nm; fluorescein glucuronide $\lambda_{\mathrm{ex}}$ 488.0 nm) and use the differences in the total fluorescence to quantify each moiety.^18^ In our work, we modified the DermaInspect^®^ to include a filter wheel [Fig. 1(d)] in which various emission bands are used in an imaging process often referred to as MPM spectral imaging. This has allowed us to keep the excitation wavelength constant and, in noninvasively imaging human skin over various emission wavelength bandwidths, to rapidly and easily acquire both z-stack and/or temporal images. An illustration of the use of spectral bandwidths to selectively quantify various endogenous and exogenous fluorophores is shown in Fig. 1(a). Here, it is observed that that the spectra of two endogenous fluorescent antioxidants in the viable epidermis, NAD(H)P and FAD, do not overlap, with $\lambda_{\mathrm{em}}$ of 440 nm (NADH-protein), 460 nm (NADH-free), and 530 nm (FAD) (Table 1), and can be imaged using a 410 to 490 nm and a 510- to 560-nm bandpass filter, respectively.^19^ Importantly, as discussed in the next section, the autofluorescence of nanoparticulate zinc oxide occurs at a lower emission wavelength, allowing it to be quantified using an emission filter with a wavelength band of 380±20  nm, without signal interference from the endogenous viable epidermal fluorescence.

**Table 1:** MPM maxima values for endogenous fluorophores of the skin and ZnO NP (recreated with permission from Ref. 17).

Component	Excitation wavelength (nm)	Emission wavelength (nm)	Fluorescence lifetime (ns)
NAD(P)H free	720 to 760	460	0.3
NAD(P)H bound	720 to 760	440	2.0 to 2.3
FAD	860 to 920	530	5.2
Keratin	720 to 800	420 to 580	0.4-2.3
Elastin	860 to 880	420 to 460	0.2/2.5
Collagen	860 to 900	430 to 450	0.3/2.0
Melanin	720 to 760, 920	440, 520, 575	0.1/1.9/8.0
ZnO NP	710 to 840	370 to 600	>0.15

The fluorescence of the viable epidermal endogenous fluorophores, also referred to as autofluorescence, allows visualization of the detailed morphological structure of the various skin layers and any changes caused by external chemicals to be seen by noninvasive multiphoton imaging. For instance, Jung et al.^20^ used MPM autofluorescence to measure viable epidermal cell density, keratinocyte size, and nucleus-to-cytoplasm ratio in stratum granulosum and stratum spinosum in the *in vivo* characterization of viable epidermal structural changes after topical application of glucocorticoids in healthy human skin.

**Fig. 2:**
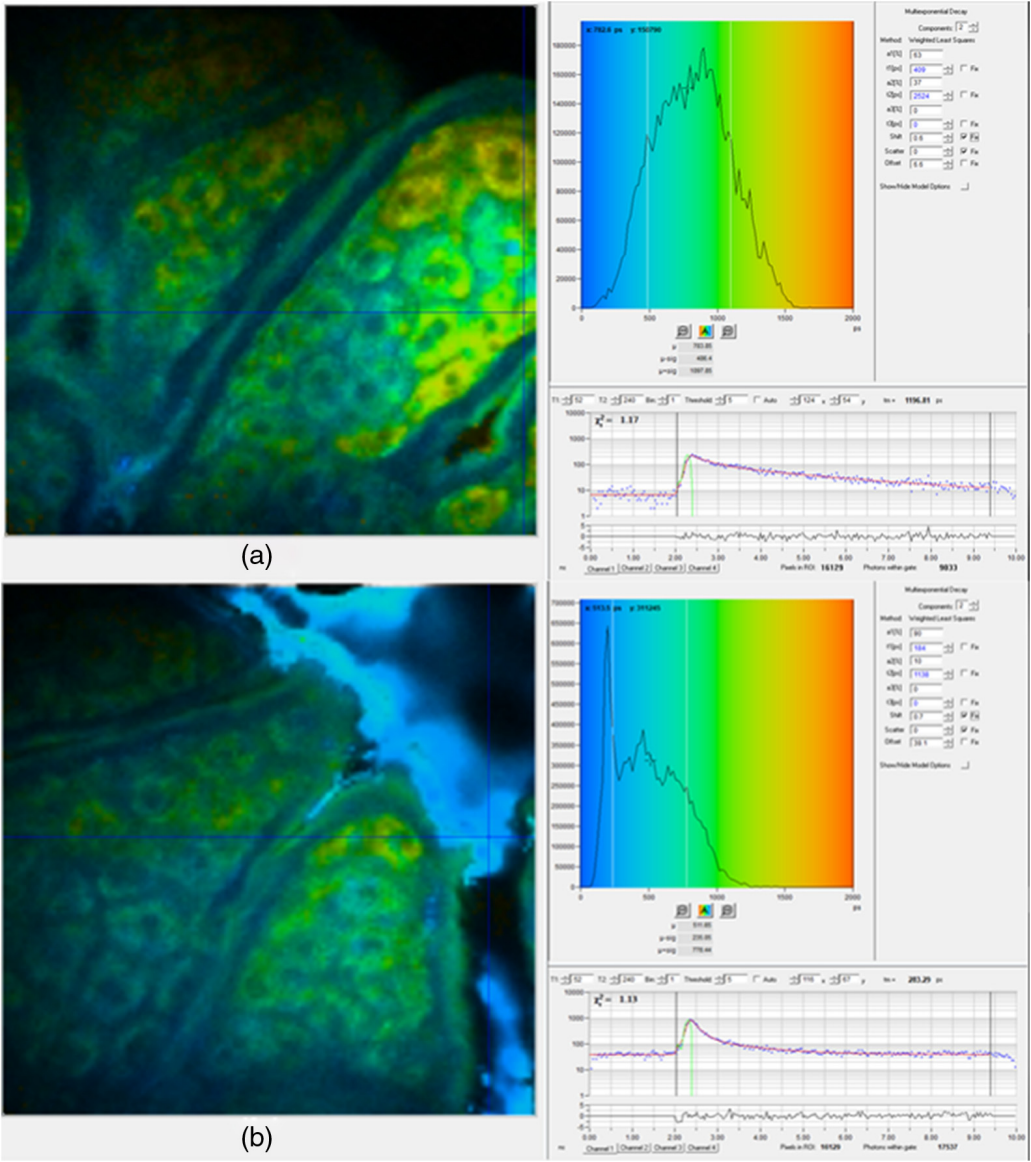
MPM of the viable epidermis determining fluorescence lifetimes of endogenous and exogenous solutes. Images of mean fluorescence lifetime, (left) $\tau_{m}$, with (right) corresponding color-coded histogram of (a) NAD(P)H in normal control skin and (b) zinc oxide nanoparticles applied to normal skin (blue regions in the furrows). (right) Graph of photon counts within (left) the pixel marked by cross-hair in the image, over time in the 10 ns between the 85-fs laser pulses.

All fluorophores have a second characteristic that may be used to characterize them and to distinguish them from other endogenous fluorophores, their fluorescence lifetime. Shown are the representative photon counts over time for NAD(P)H in the viable epidermis [Fig. 2(a)] and for zinc oxide on the stratum corneum (SC) [Fig. 2(b)] using the Mai Tai excitation laser (85-fs laser pulse width, 80-MHz repetition rate, incident optical power of 20 and 30 mW in SC and epidermis, respectively) on our DermaInspect^®^ MPM. These, in turn, can be used to define fluorescence decay curves and a mean fluorescence lifetime,^21^ which can be mapped to individual pixels within the image, as shown in Fig. 2. Various lifetimes for viable epidermal endogenous fluorophores and ZnO NPs are also shown in Table 1. It is evident that ZnO NPs have a much longer fluorescence lifetime than the various viable epidermal endogenous fluorophores; however, the fluorescence lifetime measured has been shown to be a function of the shape and surface chemistry of the ZnO NPs, which gives rise to a range of reported values from less than 1 ns up to 3  μs.^22^

The coupling of MPM with FLIM as an imaging modality has revolutionized the way one can noninvasively probe skin physiology and pharmacokinetics in health and disease. It has allowed a direct visualization of dermal structures via endogenous fluorescent biomolecules, such as keratin, elastin, melanin, porphyrins, and collagen and measurement of cellular metabolism via NAD(P)H and FAD,^1^^,^^16^^,^^23^ as well as differentiating drugs from their metabolites at a given fluorescence excitation wavelength and emission band, e.g., fluorescein from fluorescein glucuronide.^21^ In skin research, MP-FLIM has been used to investigate the metabolic status of patients with dermatitis,^24^ early detection of malignant melanoma,^25^ collagen and elastin response to cosmetic products,^26^ metabolic response to chemotherapy,^27^ and chronic wounds,^28^ as well as the accumulation of sunscreen nanoparticles in humans.^9^^–^^13^^,^^29^^–^^32^ An obvious disadvantage in having endogenous fluorophores present in human skin is that they can potentially interfere with the fluorescence from any externally applied solute. Unfortunately, only a limited number of topically applied pharmaceutical compounds have a fluorescence signal suitable for use with MPM, with zinc oxide being one. As a result, most studies involving the use of MPM in drug delivery assessments have been limited to modeling fluorescent compounds.^33^^–^^35^

## 3 Nanoparticulate Zinc Oxide Sunscreen Development and Safety Issues

In the early 2000s, the expanding field of nanotechnology provided many opportunities for pharmacological agents and adjuvants. Around the same time, companies started producing a nanoparticulate version of zinc oxide that was very appealing for sunscreen use. The formulations incorporating ZnO NPs resulted in a colorless, transparent, and aesthetically acceptable appearance when applied to the skin, unlike the chalky white film on the skin that had made zinc sunscreens unpopular among consumers. Like any new cosmeceutical ingredient, the safety and efficacy of these nanoparticles had to be established before consumer groups and regulators would endorse their use. The regulatory bodies assessing any product, including nanoparticles, objectively look at two critical aspects. To be a hazard, the substance must be both absorbed and possess inherent irritant or toxic properties.^36^ The assessed risk for a hazard estimates the probability of an adverse effect plus its severity and duration under exposure conditions that relate to local versus systemic effects for humans and the environment. Thus, studies on ZnO NP sunscreen penetration into human skin *in vivo* needed to be performed, and the possibility of damage to the local viable epidermis as well as possible penetration into the systemic circulation needed to be assessed.

Skepticism of the human use of sunscreen nanoparticles arose, in part, from the release of a report by Friends of the Earth entitled “Nanotechnology & Sunscreens: A consumer guide for avoiding nano-sunscreen.” This report used a color-coded alert system to warn consumers against nanoparticle-containing products, with selective, often unverified, and inflated interpretations of the findings to that date.^5^ At that time, we published our first *in vitro* study demonstrating that ZnO NP-containing sunscreens did not penetrate into human skin.^6^ However, these studies used aqueous receptor solutions in which ZnO NPs are essentially insoluble and an indirect determination of zinc concentrations with inductively coupled plasma-mass spectrometry was used as a measure of ZnO NP concentration. Transmission electron microscopy showed that all of the applied ZnO NPs were either on or within the superficial layers of the SC after topical application. These methods of assessing skin penetration of the nanoparticles were time consuming and ultimately limited by not being able to quantify ZnO NPs as an intact moiety. Not long after, Faunce et al.^37^ promoted a precautionary principle restricting the use of inorganic nanoparticle sunscreens, largely because there was a dearth of data proving their safety.

In addition, a pivotal confocal microscopy study entitled “Penetration of Intact Skin by Quantum Dots with Diverse Physicochemical Properties”^38^ raised questions about the safety of nanoparticles as this study showed extensive penetration of uncharged, anionic, or cationic nanoparticles down to the dermis after topical application. However, the type of skin used in the study was not defined in the title, and the findings in weanling pig skin were equated to what may happen in human skin. However, 8- to 12-week old pigs have a high follicular density,^39^ six to eight times compared with normal human skin; the follicles act as conduits for the permeability of the quantum dots. Thus, young pig skin can be considered ∼100 times more likely to take up nanoparticles than human skin, unlike small molecules, where adult pig skin have been used extensively in testing due to equivalence to human skin. Further, the quantum dots used had diameters of <12  nm, about one-third less than an individual typical ZnO NP used in sunscreens,^6^ but ZnO NPs are most often found in aggregates of ∼150  nm. Second, the quantum dots were applied to the skin in the manufacturer supplied vehicle, where the pH of solution was alkaline and may increase skin penetration of quantum dots. We then used laser-scanning fluorescence confocal microscopy to show that the neutral quantum dots (PEG-QD) penetrated into the viable epidermis of human skin after surface application to the SC at pH 8.3 but not at pH 7.0.^40^ We also found that neither the anionic or cationic quantum dots penetrated human skin at pH 7.0 nor at alkaline pHs.

## 4 Noninvasive MPM Fluorescence Demonstration that Nanoparticulate Zinc Oxide Sunscreen Does Not Penetrate Human Skin In Vivo

### 4.1 Spectral Fluorescence Imaging

**Fig. 3:**
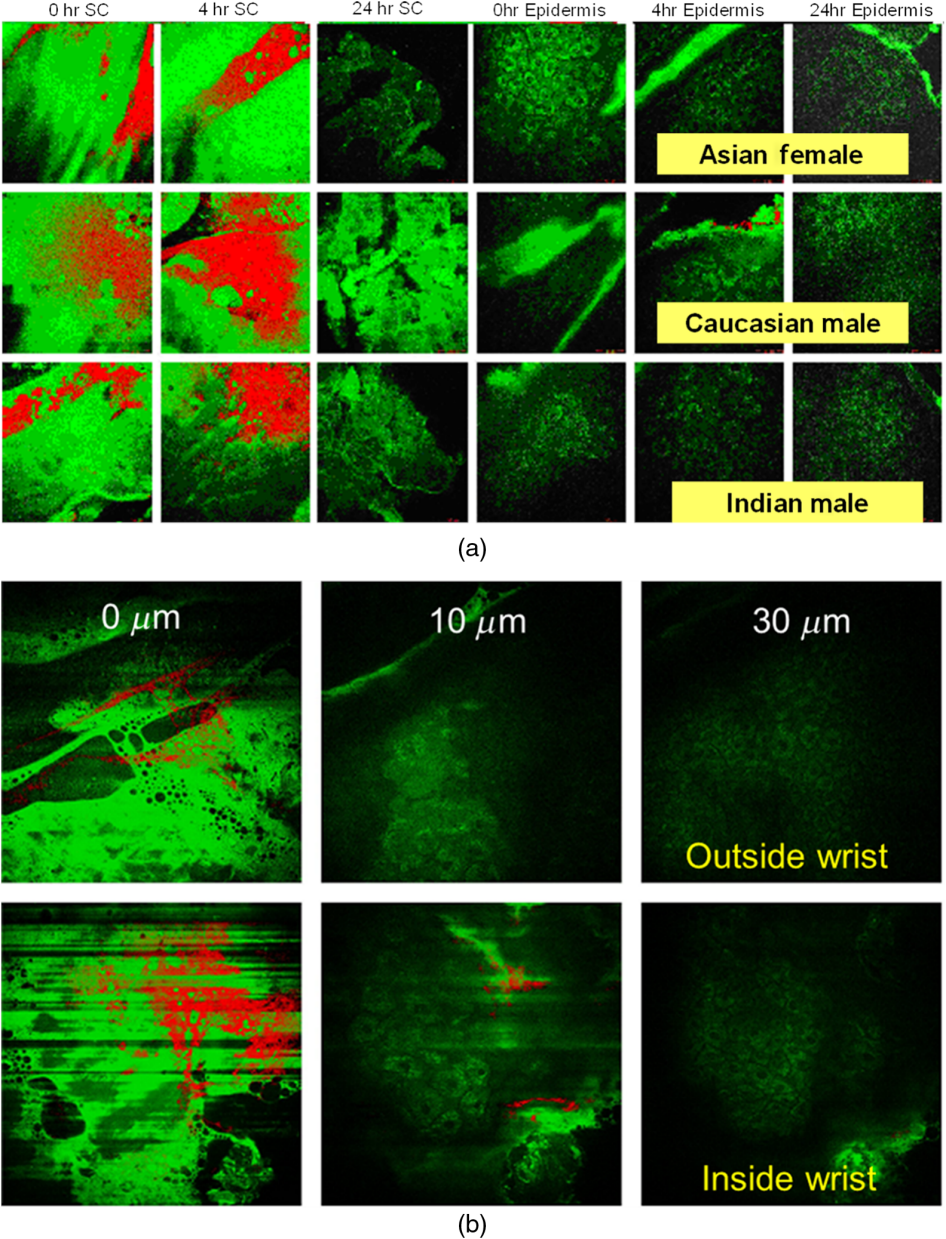
*In vivo* multiphoton spectral images of topically applied ZnO NP on human skin. (a) Observations showing skin autofluorescence (green) with ZnO NP (red) on surface SC and in furrows versus the epidermis at 0, 4, and 24 h after application to inside forearm skin for volunteers with different racial skin types (recreated with permission from Ref. 46). (b) Effect of flexing on penetration through inside and outside wrist showing ZnO NP (red) on surface (0  μm) and in furrows with no ZnO NP present in epidermis (30  μm). Note the presence of motion-related artifacts in images of inside wrist.

As mentioned earlier, our ability to perform *in vivo* imaging of zinc oxide nanoparticle penetration arose with our acquisition of a DermaInspect^®^,^16^^,^^23^ which is clinically approved in Europe for imaging skin of patients *in vivo.* Spectral fluorescence imaging was carried out with the DermaInspect^®^ equipped with a femtosecond, spectrally tunable, 80-MHz-pulsed mode-locked titanium sapphire laser (Mai Tai XF, Spectra Physics) and modified with a filter wheel, allowing imaging using a range of emission bandwidth filters. An excitation wavelength of 740 nm plus a narrow bandpass filter 380/20  nm (BP380, Omega Optical) was used to specifically quantify ZnO NPs. This was compared with data from a broad band-pass filter between 350 and 700 nm (BG39, Schott glass color filter), which captures all of the skin autofluorescence but blocks the excitation laser light.^8^ Figure 3 shows examples of our first acquired *in vivo* multiphoton spectral images of topically applied ZnO NPs on human skin.^7^^,^^8^ Applied ZnO NPs remain on the skin surface or are deposited in skin furrows after application to the inside forearm skin of volunteers with different racial skin types [Fig. 3(a)]. The effect of flexing on ZnO NP penetration through skin on the inside and outside wrist, again, shows that ZnO NPs remained on the skin surface and in furrows [Fig. 3(b)], but these results are unpublished due to the considerable *in vivo* motion-related artifacts because of the relatively long acquisition times of spectral imaging, which was later resolved using MP-FLIM.^10^^,^^41^ It is important to recognize that the spectral imaging method, as we introduced it, has limitations in sensitivity and selectivity at low ZnO NP concentrations when the overlapping signal from autofluorescence is overwhelming, especially from keratin, which spectrally overlaps with the ZnO NP.

### 4.2 Fluorescence Lifetime Imaging Microscopy

In our early MPM studies with zinc oxide, we measured its fluorescence lifetime and found that it was somewhat longer than that of the endogenous fluorophores (Fig. 2; Table 1), as well as having a very high fluorescence intensity. We therefore sought to take advantage of the distinctive ZnO NP fluorescence differences in intensity, emission spectrum, and decay profile, recognizing that quantifying a fluorescence decay requires a fluorescence intensity detector that can measure at a resolution on the picosecond time scale. In this work, we used the Becker and Hickl time-correlated single photon counting (TCSPC) system where an FLIM image is based on the different arrival times of single photons for different fluorescent solutes after a given excitation pulse (here at 740 nm) and building up a photon distribution for each pixel in the scanned area.^21^ We modeled our FLIM data assuming a double exponential decay profile:

$$f(t)=a_{1}e^{-t/\tau_{1}}+a_{2}e^{-t/\tau_{2}}\;\text{with }a_{1}+a_{2}=1,$$

where $a_{1}$ and $a_{2}$ are the amplitudes and $\tau_{1}$ and $\tau_{2}$ are the fluorescence lifetimes of the fast and slow decay components, respectively. The instrument response function of each FLIM image is convoluted with the model function, f(t), to obtain the function F(t). The function F(t) is fitted to each measured pixel to calculate and create a color-coded image of each decay parameter. In the ZnO NP analysis, a band pass filter was used to detect ZnO NPs and NAD(P)H (350 to 450 nm), whereupon the fast decay component $\tau_{1}$ from 0 to 500 ps was attributed to NAD(P)H and ZnO NPs, whereas $\tau_{2}$ from 0 to 5000 ps was attributed to NAD(P)H. The MP-FLIM decay curve of ZnO NP is shown in Fig. 2. This study also established TCSPC as the methodology of choice when examining the characteristic fluorescence decay function of skin and nanoparticles. In addition, this method overcame the issues of motion artifact often seen in spectral imaging due to the change from relatively long acquisition times to image acquisition times of under 10 s with MP-FLIM.

**Fig. 4:**
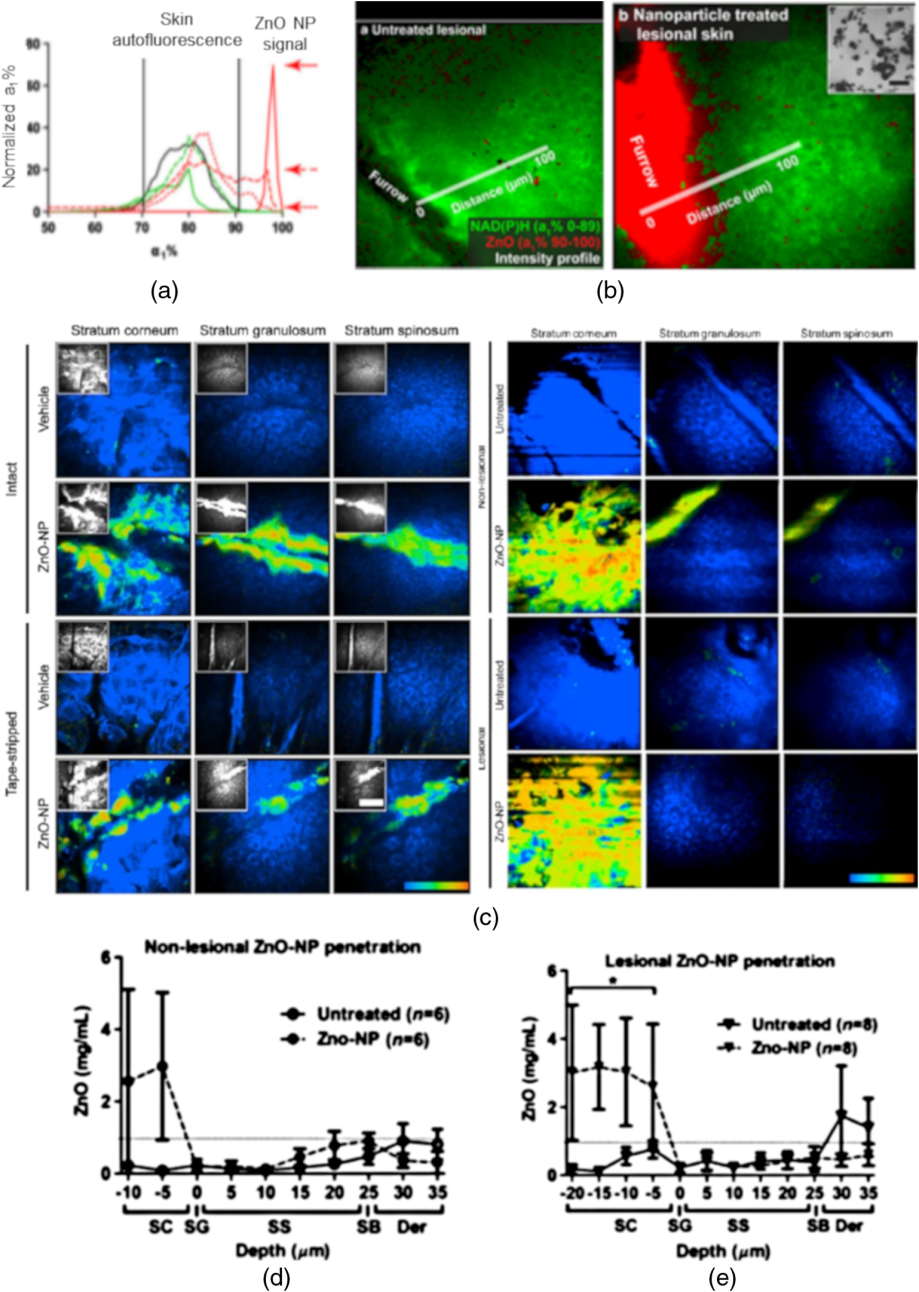
(a) Distinct $a_{1}\%$ signals from skin autofluorescence and ultrafast ZnO NP MEP. (a) The solid red line and red arrows indicate the maximal ZnO NP intensity of ZnO NP alone and after 4 and 24 h on the surface of the skin. (b) (left) Images of ZnO NP zinc oxide nanoparticles untreated lesional psoriatic skin, (right) autofluorescence (green) and ZnO NP (red) aggregate in skin furrows with of sunscreen-treated lesional sites based on $a_{1}\%$. The inset (in right panel) is an electron microscopy image of the individual ZnO NP with a mean diameter of 35 nm. Scale bar is 100 nm (adapted with permission from Ref. 42). (c) *In vivo* MP-FLIM images of intact, tape-stripped, nonlesional, and lesional volunteer skin at different depths after 24 h treatment.^12^ These color images depict the autofluorescence as blue ($a_{1}\%$ 0 to 85) and ZnO NP as green-red ($a_{1}\%$ 90 to 100) in volunteer skin. The gray scale insets are intensity-only images. Scale bar is 100  μm. (d), (e) ZnO NP penetration profiles, excluding furrows, in (d) nonlesional and (e) lesional volunteer skin, using $a_{1}\%$ 90 to 100 normalized to the region of interest area to quantify ZnO NP signal (adapted with permission from Ref. 12). *p value of >0.005 when compared with untreated skin.

TCSPC FLIM exponential decay data can alternatively be used to determine the signal from ZnO NPs, where values of $a_{1}\%$ from 90 to 100 are attributed to the ultrafast ZnO NP MEP [Fig. 4(a)]. The $a_{1}\%$ from 0 to 85 arise mainly from untreated NAD(P)H and keratin, which occurs as a relatively slower MEP signal^42^ [Fig. 4(b)]. Lin et al., using MP-FLIM MEP analysis, showed that ZnO NPs did not penetrate normal and simulated barrier impaired skin (tape stripped, as a model for sunburn), and moreover, did not penetrate lesional or nonlesional skin of psoriasis patients, [Figs. 4(c)–4(e)].

We have also used MP-FLIM to show that there was no skin penetration of gold nanoparticles^43^ and MPM localized surface plasmon resonance signal to demonstrate no skin penetration of silver nanoparticles.^44^

### 4.3 Second Harmonic Generation

Both MP-SHG/HRS and MP-FLIM have been used to quantify the concentration of ZnO NPs in the applied sunscreen with the use of standard curves and have resulted in similar resolutions of ZnO NPs. Autofluorescence arising from the skin needs not overlap the signal of interest from ZnO NPs, separated by spectral filters, fluorescence lifetimes, and physical location. At an excitation wavelength of 760 nm, SHG radiation from the ZnO NPs can be detected at half the excitation wavelength (380 nm) quasi-instantaneously with a narrow spectral width and separated from the autofluorescence of the collagen-rich dermis, which appears at much higher emission wavelengths for this same excitation wavelength. We generated an SHG/HRS from ZnO NPs on a nonlinear optical microscope by excitation of samples at 770 nm and then used specific narrow band filters and direct imaging of ZnO NPs to show that ZnO NPs do not penetrate human skin.^45^ FLIM can be enhanced using TCSPC in conjunction with SHG to identify the ZnO NPs.

Darvin et al.^14^ used spectral tuning to demonstrate ZnO NP SHG/HRS. They showed that the signal that prominent emission peak was always half the excitation wavelength used to excite ZnO NPs and that an excitation of 760 nm was optimal. They further suggested that the two-photon-induced fluorescence we had previously observed can be neglected. They went on to use their SHG/HRS method to conclude that, with a detection limit of $<0.08\;\mathrm{fg}/\mu m^{3}$ and a signal-to-noise ratio of ∼10, ZnO NPs penetrate only into the outermost layers of SC in furrows, and into the orifices of the hair follicles and do not reach the viable epidermis. This paper claimed an ∼100-fold greater sensitivity than we had reported; however, the calculations, presented in a cubic micron volume as opposed to our reported sensitivity in a two-dimensional focal plane, are in fact very similar. Our estimated sensitivities, measured on a glass slide, were in the ranges of 0.51 to 1.2 and 0.13 to 0.5  mg/mL for coated and uncoated ZnO NPs, respectively.

It is important to recognize that the reason why ZnO NPs do not penetrate the human skin is that they do not exist as individual nanoparticles per se but, especially in sunscreen products, are bound together as porous aggregate clusters, as we have shown in our scanning electron micrography characterization of ZnO NPs.^6^ As a consequence, these ZnO NP aggregates differ from the larger white ZnO particles in retaining the transparency conferred by being formed from ZnO NPs. In practice, ZnO NPs used in sunscreens are either uncoated or coated with silicone derivatives, such a triethoxycaprylylsilane or other agents. Coated ZnO NPs enable easier mixing with other ingredients and easier and more even spreading of the resulting ZnO NPs on the skin and, in principle, are less photoreactive. Such coating may also protect the luminescent centers of ZnO NPs.^22^ Our work has also shown that these coatings may affect ZnO NP fluorescence lifetimes. We found that the uncoated ZnO NPs have a mean $\tau_{1}$ lifetime of 150 ps and a $\tau_{2}$ lifetime of 860 ps whereas coated ZnO NPs have a mean $\tau_{1}$ lifetime of 250 ps and a $\tau_{2}$ lifetime of 1400 ps.^9^ Darvin et al. also claimed to be able to distinguish between the particulate (ZnO NP) and dissolved (${\mathrm{Zn}}^{++}$) forms of Zn but presented no information on any analytical results that they had obtained for dissolved zinc ions.

### 4.4 Phosphorescence Lifetime Imaging Microscopy Measurements via Photoluminescence Offset

**Fig. 5:**
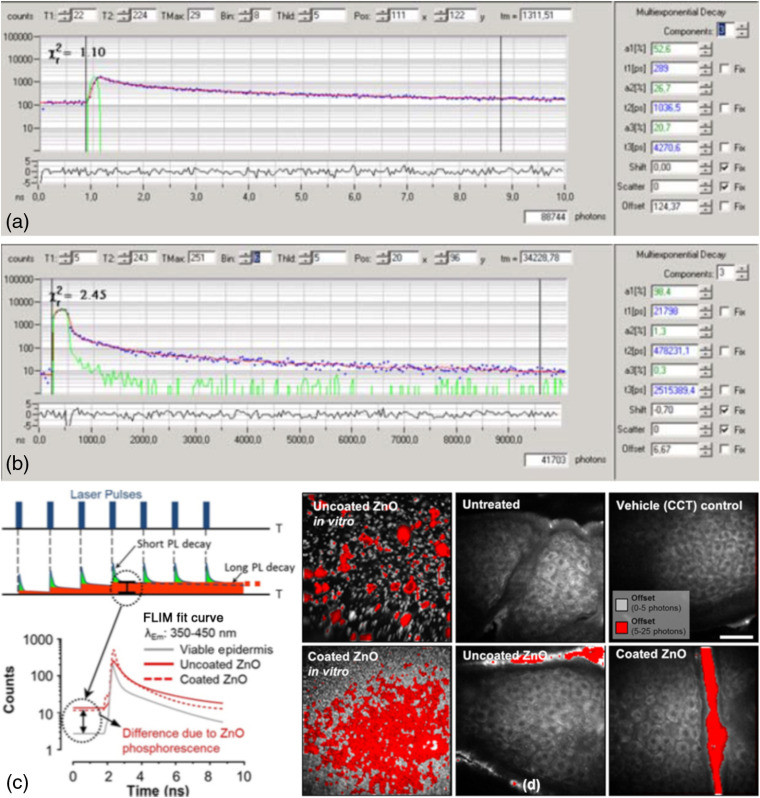
Fluorescence decay fit curves of ZnO NP: (a) fast decaying photoluminescence collected over 10 ns and (b) direct lifetime measurement using phosphorescence lifetime imaging (PLIM) collected over 10  μm. Reproduced with permission. (c) The enhanced background signal seen at time=0  ns is related to the ZnO NP concentration where the signal has accumulated due to the repeated laser pulsing to create an “offset” that results in a more sensitive multicomponent fit of the data. The multicomponent fit of PLIM is less sensitive because there is loss of photons over a longer period, up to 10  μs when the decay returns to background conditions. (d) Schematic of the ZnO NP lifetimes as quantified by a quasicontinuous accumulation of steady-state photoluminescence. (d) Representative multiphoton images of uncoated and coated ZnO NP *in vitro* and topically applied to *in vivo* human skin for 6 h for signal isolation and quantification. “Offset” values <5 counts are shown in grayscale and include cellular autofluorescence, while “offset” values above the autofluorescence- “offset” level were assigned to ZnO NP (red). Scale bar=40  μm. With permission from Refs. 11 and 46.

The need to improve specificity as well as the detection limit of the ZnO NP signal was a strong motivation to develop newer and more reliable methods. In our early studies, we showed that uncoated and coated ZnO NPs had very long phosphorescence lifetimes (Fig. 5). These phosphorescence lifetimes for ZnO NPs are incompatible with imaging using FLIM because phosphorescence is a radiative relaxation mechanism, which takes place over a much longer time scale than fluorescence so that its emission is incomplete when the next laser excitation pulse arrives in the usual FLIM modeling. Becker & Hickl applied this finding to create their FLIM/phosphorescence lifetime imaging microscopy (PLIM) system, which can modulate on/off synchronously the high-frequency-pulsed laser beam used to scan a sample.^45^ Whereas FLIM data are acquired during scanning with the usual series of FLIM excitation pulses, PLIM is a slower process, requiring both a continuous accumulation of phosphorescence to quasisteady state through repeated pulsing of the ZnO NP during this initial FLIM phase followed by a phosphorescence decay when the pulsing is stopped over milliseconds. The Becker & Hickl device modulates the laser to both acquire the phosphorescence for materials of interest during the ON FLIM phase and then turns off the laser at a defined time to record phosphorescence imaging count rates for at least 100  μs, much longer than the usual 0.01  μs used for MP-FLIM imaging.^44^^,^^47^ While this commercialized PLIM system has been used in a number of studies,^48^ including unpublished *in vitro* skin studies on our Adelaide Zeiss 710 instrument, we are not aware of any studies using the commercial FLIM/PLIM device for *in vivo* human skin imaging.

Given that the commercial FLIM/PLIM device was not available on our Brisbane DermaInspect^®^ instrument, we sought an alternative solution to quantifying ZnO NP by its phosphorescence using *in vivo* human skin imaging. The commercial FLIM/PLIM device measures the PLIM decay after turning off the repeated laser pulses once the steady-state photoluminescence has been reached. What we realized is that this steady-state photoluminescence will be an additional background noise to that seen before the laser excitation of ZnO NP that is also a means of quantifying ZnO NP, as shown in Fig. 5(c). Here, it is seen that the ZnO NP slow decaying photoluminescence accumulates as an enhanced background signal and can be quantified as a measurable “offset” in the recorded decay background. This amount of offset, in turn, can then be used as a measure of the amount of ZnO NPs present in the tissue when adjusted for the cellular autofluorescence [Fig. 5(d)].

## 5 Using MPM to Address Consumer Concerns about Safety of Nanoparticulate Zinc Oxide

A number of very thoughtful articles have argued that nanoparticulate sunscreens should not be used until we have resolved a number of hitherto unanswered questions, such as how safe are these sunscreens when applied under “in use conditions.”^5^^,^^37^^,^^49^ They promote a precautionary principle, which restricted the use of inorganic nanoparticle sunscreens until there was sufficient data to prove their safety.

**Fig. 6:**
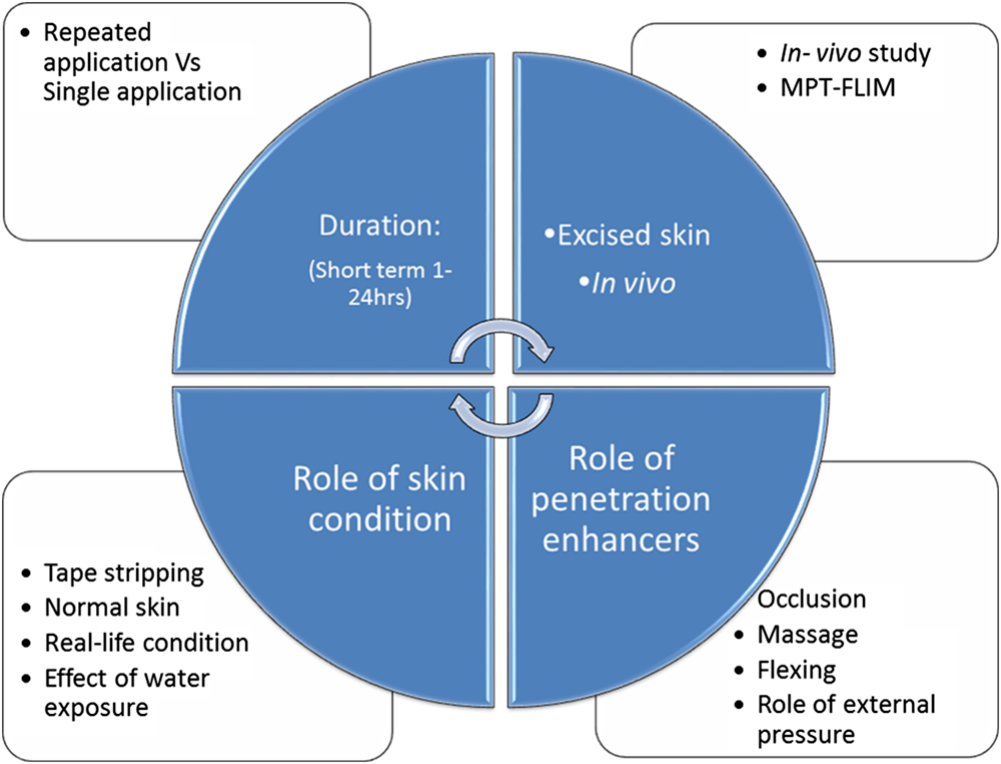
Assessment of nanoparticulate sunscreens. Drug, drug product, and skin conditions.

**Fig. 7:**
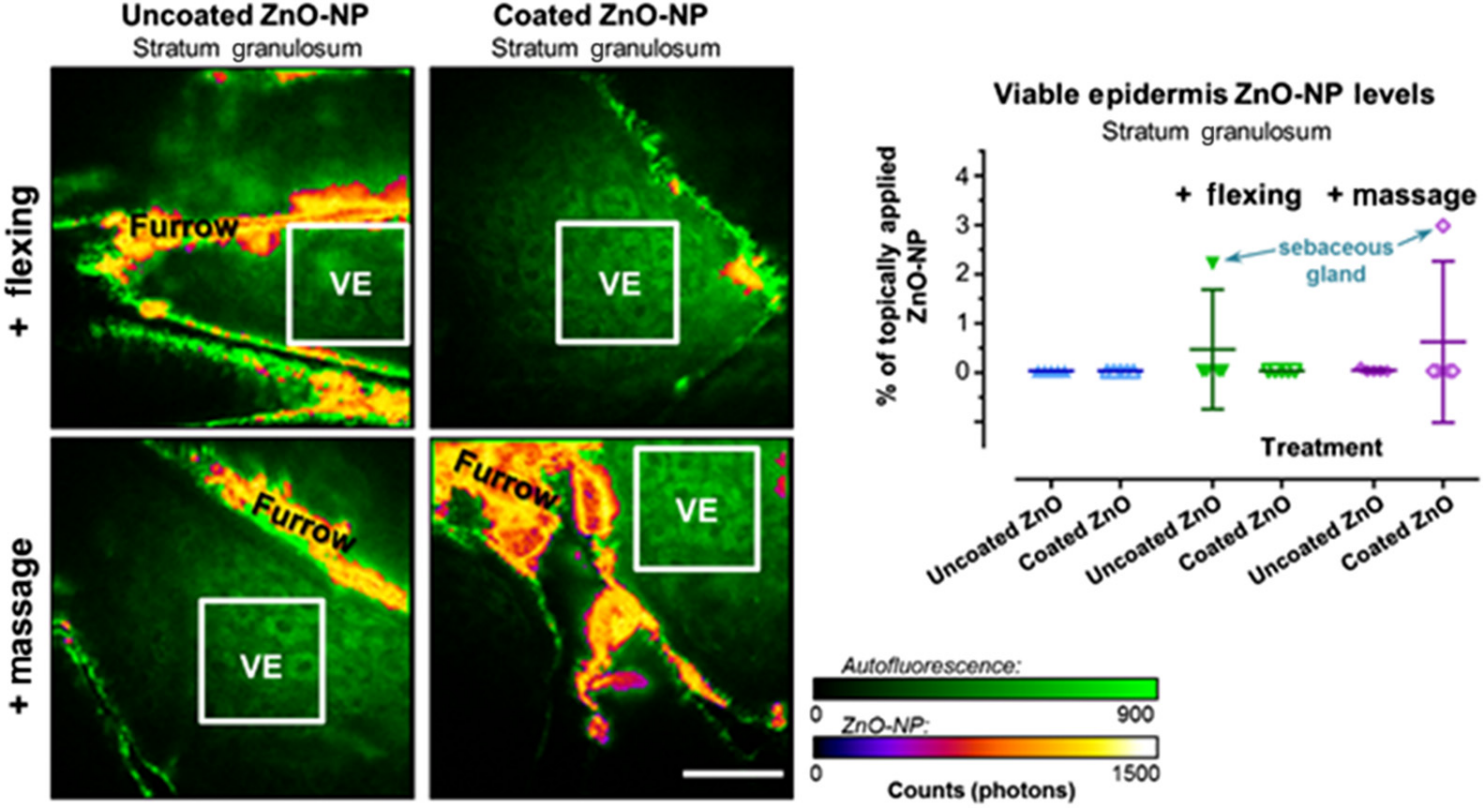
Penetration of topically applied uncoated and coated ZnO NP within the stratum granulosum layer of human skin *in vivo* following application with flexing and massage. (a) Representative FLIM images of SG of human skin following flexing (top) and massage (bottom). Images are pseudocolored by offset values to distinguish cellular autofluorescence (green) and ZnO NP signal of the long luminescence component (violet-yellow-white). Scale bar=40  μm. (b) Quantification of ZnO NP (% of applied dose) in regions of interest in the SG of all volunteers (n=5). Used with permission from Ref. 10.

We systematically sought to apply our MPM technologies to quantifying the penetration of ZnO NPs into human skin to address the specific examples raised by Friends of the Earth and Faunce et al., including how safe is it to apply nanoparticulate zinc oxide to flexed, occluded, damaged, and diseased skin in multiple dose studies. Figure 6 summarizes the various conditions that were mapped in these studies. One research approach was to try to match the formulations of products currently on the market and apply protocols that would inform “in use conditions.” Hence, three formulations representing the most abundant classes of commercial topical products were batch manufactured at laboratory scale, and ZnO NPs were suspended in these vehicles at 10% (w/w) and then assessed. One of the highlights of this work was that the concentrations of ZnO NPs in the furrows and viable epidermal cellular region was semiquantitatively assessed and reported.^13^ A greater concentration of ZnO NP (>eightfold) could be detected in the furrows when compared with the surface of the SC. As it has been claimed that furrows can act as reservoirs for dissolved drugs and small molecules,^50^ this observation is highly significant. However, nanoparticles, even those that accumulate in furrows, have been demonstrated to be easily washed off even from wounded skin.^51^ Another important concern arose from studies with beryllium particles, which illustrated that skin flexing could loosen SC and thereby pose a penetration risk.^52^ This work informed the massage and flexing studies that we undertook to assess their possible effect on enhancement of nanoparticle penetration. Hence, a customized study was designed to explore the effects of massage and skin flexing on ZnO NP penetration during topical product application and day-to-day activities.^10^ Figure 7 shows how flexing and massage caused no nanoparticle penetration, with the two outlier values known to be measurements from sebaceous glands rather than deeper viable skin layers. We concluded that there was no evidence for the physical forces generated by massage and flexing, routinely met in normal sunscreen use, to enhance the skin penetration of ZnO NP. We also studied the effects of repeated treatment on ZnO NP penetration in human volunteers. MPM and MP-FLIM once again demonstrated a lack of ZnO NP penetration after (1) six hourly repeated applications within a day and (2) daily application for 5 consecutive days. Simultaneous SHG detection of the intact nanoparticles on the skin surface conclusively verified no nanoparticle penetration.^13^

## 6 Using MPM to Show Conversion of Nano Zinc Oxide to Zinc Ions and Their Absorption into Viable Epidermis

**Fig. 8:**
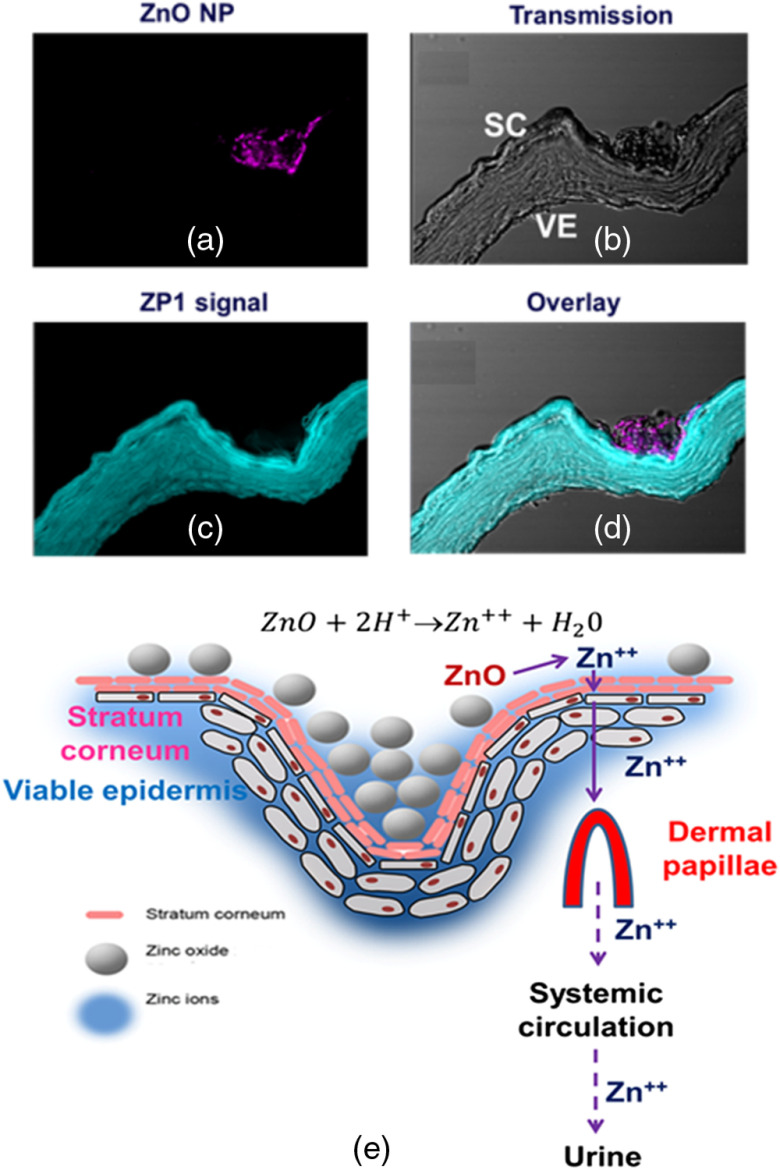
MP-SHG and MP-FLIM images of cryosection *ex vivo* human skin after 48 h of applied sunscreen containing ZnO NPs followed by product removal. (a) MP-SHG signal for ZnO NPs (pink) had accumulated within the skin furrow. (b) Transmission image of skin labeled with SC for SC and VE for viable epidermis (gray). (c) MP-FLIM ZP1 fluorescence signal (blue-green). (d) Overlay of all channels. (e) Conversion of zinc oxide to zinc ions on the surface of the skin or in the SC and relative penetration into viable epidermis. Reprinted with permission from Ref. 30.

Akin to the efforts to directly image the location of applied ZnO NPs, we also sought to directly image the location of zinc ions relative to the ZnO NPs. For this, we employed ZinPyr-1 (ZP1), a dye that fluoresces in the presence of labile zinc. Figure 8 shows an example of our imaging studies in which we simultaneously imaged the ZnO NPs and ZP1–zinc complex after application of ZnO NPs to *ex vivo* human skin followed by product removal. It is evident that ZnO NPs had accumulated on the skin surface within a skin furrow when measured by their SHG (excitation at 800 nm and emission at 370 to 410 nm) [Fig. 8(a)]. However, there was no detectable SHG signal within the viable epidermis, demonstrating that the ZnO NPs do not cross the skin barrier. Figure 8(b) validates the presence of ZnO NPs in the furrows. Importantly, there is a natural residual amount of ZP1–zinc complex in the viable epidermis, which is also seen in control skin (ZP1–zinc complex excitation at 488 nm and emission at 520 to 560 nm) [Fig. 8(c)]. Figure 8(d) shows that the intensity of this ZP1–zinc complex is much brighter after application of ZnO NPs to the skin, especially adjacent to the furrows where the ZnO NPs are found, confirming zinc ion penetration into the viable epidermis after its formation on the breakdown of nanoparticulate zinc oxide. The process of conversion of zinc oxide to zinc ions and absorption into the viable epidermis and thence into the body is shown in Fig. 8(e).

Our findings are significant in the context of two other publications using zinc ions as a surrogate measure for ZnO NP penetration of human skin. While Cross et al. reported that no ZnO NPs penetrated beyond the outer skin SC surface, using electron microscopy, they found that nonsignificant, low concentrations of zinc (∼0.03% of applied dose) had permeated across the isolated human epidermis. Consistent with their findings, Gulson et al. reported a small increase in ^68^Zn within the blood and urine that increased over 5 days after topical application of the stable isotope^68^ Zn-enriched ZnO NPs on human skin under “in use conditions.”^53^^,^^54^ Our MPM findings using SHG detection for ZnO NP for ZP1–labile zinc complexes (Fig. 8) show that only solubilized zinc penetrates to the viable epidermis after topical ZnO NP application and the amount of zinc absorbed is extremely low relative to the baseline level of zinc in the skin.

## 7 Characterizing Viable Epidermal Morphology and Redox State by MPM in Assessing the Safe Use of Topical Zinc Oxide Nanoparticles

**Fig. 9:**
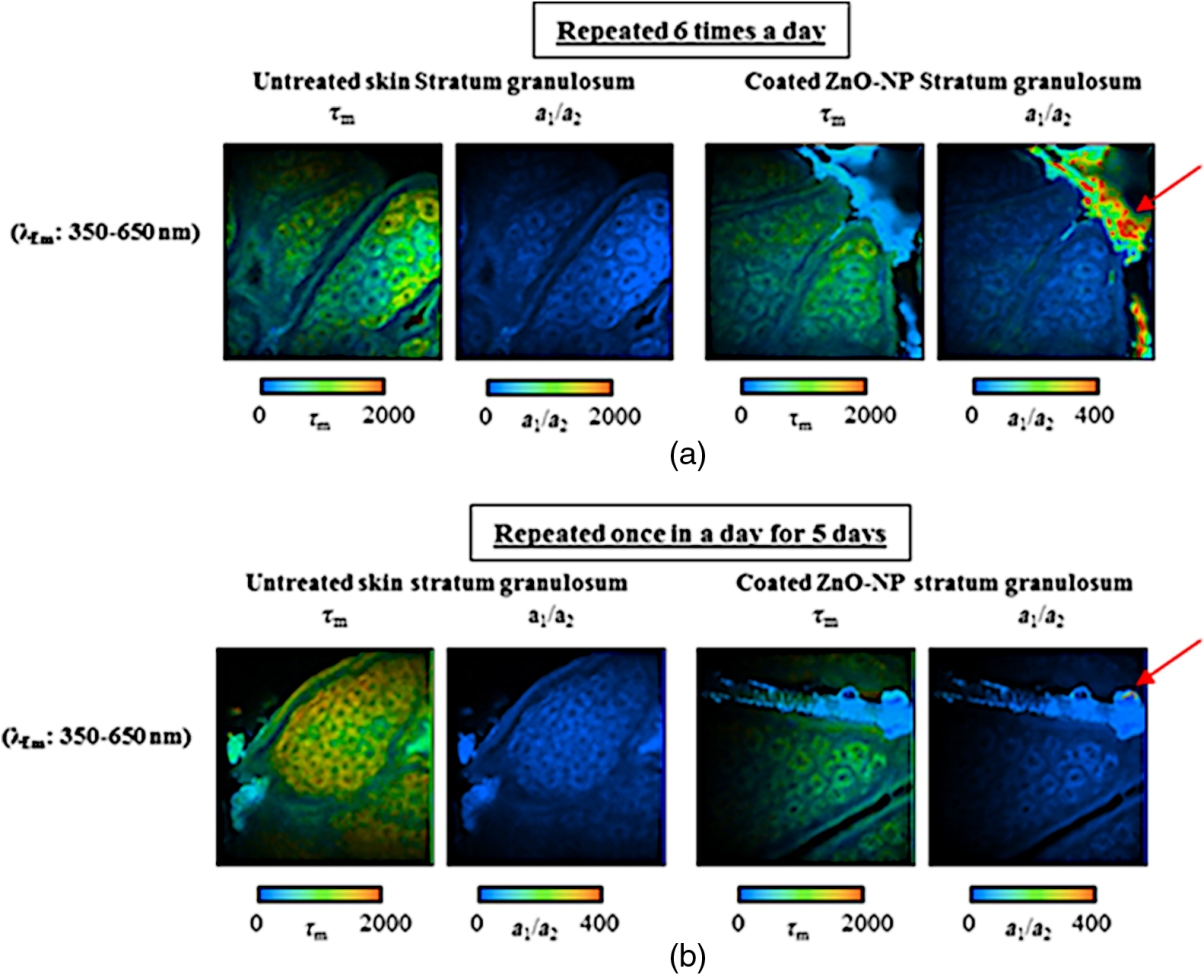
*In vivo* NAD(P)H average lifetimes of $\tau_{m}$ and $a_{1}/a_{2}$ of human skin either untreated or treated with coated ZnO NPs (a) repeated six times a day and (b) repeated once a day for five days, demonstrate no change in the cells of the stratum granulosum. The ZnO NPs in the furrow is seen to possess a higher $a_{1}/a_{2}$ ratio (red arrow). Data from Ref. 13 reanalyzed.

In 2011, we began using MP-FLIM to simultaneously analyze the fluorescence lifetime behavior of NAD(P)H and FAD as metabolic markers of the viable epidermal cellular redox state and health in relation to potential adverse effects caused by ZnO NP in human *in vivo* skin.^8^^–^^13^ This work was important as Sharma et al. and Akhtar et al. had previously reported that *in vitro*, the preliminary mode of ZnO NP toxicity on human liver cells and human cancer, is due to selectively induced apoptosis and ZnO NPs are able to induce DNA damage on human epidermal keratinocytes,^55^^–^^57^ but this has not been shown to be true *in vivo*. We and others have demonstrated that apoptosis is associated with an increase in the mean fluorescence lifetime of NAD(P)H and a decrease in the free-to-bound NAD(P)H ratio.^58^^–^^60^ Hence, using the same principles of metabolic analysis, we have demonstrated that, after treatment with ZnO NPs, the occurrence of normal fluorescence lifetimes in the skin epidermal region is a sign of the normal metabolic state of the cells and lack of penetration of ZnO NPs. Figure 9 shows the average mean fluorescence lifetime of NAD(P)H in untreated and ZnO NP-treated human volunteer skins from experimental data of our repeated application of ZnO NPs study.^13^ Had the nanoparticles penetrated and reached the epidermal cells at high enough concentrations to cause apoptosis, our sensitive analytical techniques would have been ideally suited to record a change in the metabolic indicators.

## 8 Conclusion

After a decade of using MPM to show the negligible human skin penetration of mineral nanoparticle sunscreens, these UV protective agents are now starting to be promoted ahead of the traditionally used organic sunscreens. Recently, a small randomized clinical trial of 24 healthy volunteers was conducted to determine bloodstream concentrations of four active ingredients (avobenzone, oxybenzone, octocrylene, and ecamsule) in four over-the-counter cream, lotion, or spray formulations of chemical sunscreens.^61^ It was found that all of the products resulted in plasma concentrations of the active ingredients that triggered a requirement by the FDA for further systemic safety testing. The limitations of this study are that the sunscreens were not exposed to heat, sunlight, humidity, or normal activity and did not include a range of skin types, which may affect the rate of sunscreen absorption. Subsequently, the FDA has published proposed rules for sunscreen drug products for over-the-counter human use (21 CFR § 201, 310, 347, and 352 2019) and have recommended that zinc oxide and titanium dioxide-based sunscreen products containing up to 25% these ingredients are safe for human use. Sunscreens with active ingredients aminobenzoic acid (PABA) and trolamine salicylate have risks that outweigh their benefits. Further testing is required for the use of cinoxate, dioxybenzone, ensulizole, homosalate, meradimate, octinoxate, octisalate, octocrylene, padimate O, sulisobenzone, oxybenzone, and avobenzone.

In conclusion, the culmination of our studies of noninvasively imaging the penetration of nanoparticulate zinc oxide across normal and disordered human skin under various “in use conditions” with MPM has shown that ZnO NPs are unlikely to penetrate the outermost layer of the skin, the SC. This has, in turn, allowed us to help answer the pressing and important toxicological question of nanoparticle sunscreen safety. This was only possible through the foresight and perseverance of Karsten König and his team at JenLab in creating and obtaining the CE registration for the DermaInspect^®^ to image human skin, as well as the important ingenious contributory work of Wolfgang Becker in creating the TCPSC FLIM system to provide us with the technology that has underpinned our work. We have been fortunate to have the expertise and interest to apply this MPM technology to better understand the safety of ZnO NPs. This has led us to use six different MPM methods for ZnO NP detection over more than ten years in our quest to document the safety, or otherwise, of ZnO NP-based sunscreens. As it turns out, with the recent concerns raised about organic sunscreens, our work may have been timely for the beach going and sun-seeking consumer who now has access to recommended zinc oxide or titanium dioxide-based sunscreens that are their best protection from sunburn, photoaging, and future skin cancers.

## References

[1] König K. et al., “Applications of multiphoton tomographs and femtosecond laser nanoprocessing microscopes in drug delivery research,” Adv. Drug Delivery Rev. 63, 388–404 (2011). ADDREP 0169-409X 10.1016/j.addr.2011.03.002 21514335

[2] AAD, “American Academy of Dermatology Association: skin cancer,” 2019, https://www.aad.org/media/stats/conditions/skin-cancer (accessed 24 8 2019).

[3] Sunscreen RM, “Product specifications—zinc mineral-based lotion,” 2019, https://www.rmsunscreen.com/productspecs.aspx (accessed 24 8 2019).

[4] White I. R., “The scientific committee on cosmetic products and non-food products intended for consumers,” European Commission SCCP, Brussels (2003).

[5] Berube D. M., “Rhetorical gamesmanship in the nano debates over sunscreens and nanoparticles,” J. Nanopart. Res. 10, 23–37 (2008). JNARFA 1388-0764 10.1007/s11051-008-9362-7

[6] Cross S. E. et al., “Human skin penetration of sunscreen nanoparticles: *in vitro* assessment of a novel micronized zinc oxide formulation,” Skin Pharmacol. Physiol. 20, 148–154 (2007). 10.1159/000098701 17230054

[7] Roberts M. S., “The latest science (including safety) on nanotechnology and skin penetration,” in FDA Public Meeting on Nanotechnology (2006).

[8] Zvyagin A. V. et al., “Imaging of zinc oxide nanoparticle penetration in human skin *in vitro* and *in vivo*,” J. Biomed. Opt. 13(6), 064031 (2008). JBOPFO 1083-3668 10.1117/1.3041492 19123677

[r9] Leite-Silva V. R. et al., “The effect of formulation on the penetration of coated and uncoated zinc oxide nanoparticles into the viable epidermis of human skin *in vivo*,” Eur. J. Pharm. Biopharm. 84, 297–308 (2013). EJPBEL 0939-6411 10.1016/j.ejpb.2013.01.020 23454052

[r10] Leite-Silva V. R. et al., “Effect of flexing and massage on *in vivo* human skin penetration and toxicity of zinc oxide nanoparticles,” Nanomedicine 11, 1193–1205 (2016).1743-5889 10.2217/nnm-2016-0010 27102240

[r11] Leite-Silva V. R. et al., “Human skin penetration and local effects of topical nano zinc oxide after occlusion and barrier impairment,” Eur. J. Pharm. Biopharm. 104, 140–147 (2016). EJPBEL 0939-6411 10.1016/j.ejpb.2016.04.022 27131753

[r12] Lin L. L. et al., “Time-correlated single photon counting for simultaneous monitoring of zinc oxide nanoparticles and NAD(P)H in intact and barrier-disrupted volunteer skin,” Pharm. Res. 28, 2920–2930 (2011). PHREEB 0724-8741 10.1007/s11095-011-0515-5 21717255

[r13] Mohammed Y. H. et al., “Support for the safe use of zinc oxide nanoparticle sunscreens: lack of skin penetration or cellular toxicity after repeated application in volunteers,” J. Invest. Dermatol. 139, 308–315 (2019). JIDEAE 0022-202X 10.1016/j.jid.2018.08.024 30448212

[14] Darvin M. E. et al., “Safety assessment by multiphoton fluorescence/second harmonic generation/hyper-Rayleigh scattering tomography of ZnO nanoparticles used in cosmetic products,” Skin Pharmacol. Physiol. 25, 219–226 (2012). 10.1159/000338976 22653438

[15] Fischer F. et al., “Assessing the risk of skin damage due to femtosecond laser irradiation,” J. Biophotonics 1, 470–477 (2008). 10.1002/jbio.200810050 19343673

[16] Helm P. J. Ottersen O. P., “Proposal of a new method to measure FRET quantitatively in living or fixed biomedical specimens on a laser microscope,” Proc. SPIE 7903, 790331 (2011). 10.1117/12.874073

[17] Holmes A. et al., “Revealing interaction of dyes and nanomaterials with organs by imaging,” Chapter 18 in Multiphoton Microscopy and Fluorescence Lifetime Imaging Applications in Biology and Medicine, König K., Ed., pp. 345–368, DeGruyter Berlin, Germany (2018).

[18] McLaren J. W. Brubaker R. F., “Measurement of fluorescein and fluorescein monoglucuronide in the living human eye,” Invest. Ophthalmol. Vision Sci. 27, 966–974 (1986). 3710736

[19] Huang S. Heikal A. A. Webb W. W., “Two-photon fluorescence spectroscopy and microscopy of NAD(P)H and flavoprotein,” Biophys. J. 82, 2811–2825 (2002). BIOJAU 0006-3495 10.1016/S0006-3495(02)75621-X 11964266 PMC1302068

[20] Jung S. et al., “*In vivo* characterization of structural changes after topical application of glucocorticoids in healthy human skin,” J. Biomed. Opt. 22(07), 076018 (2017). JBOPFO 1083-3668 10.1117/1.JBO.22.7.076018 28753693

[21] Roberts M. S. et al., “Non-invasive imaging of skin physiology and percutaneous penetration using fluorescence spectral and lifetime imaging with multiphoton and confocal microscopy,” Eur. J. Pharm. Biopharm. 77, 469–488 (2011). EJPBEL 0939-6411 10.1016/j.ejpb.2010.12.023 21256962

[22] Zhang Z.-Y. Xiong H.-M., “Photoluminescent ZnO nanoparticles and their biological applications,” Materials 8, 3101–3127 (2015). MATEG9 1996-1944 10.3390/ma8063101

[23] Koenig K. Riemann I., “High-resolution multiphoton tomography of human skin with subcellular spatial resolution and picosecond time resolution,” J. Biomed. Opt. 8(3), 432–439, 438 (2003). JBOPFO 1083-3668 10.1117/1.1577349 12880349

[24] Huck V. et al., “From morphology to biochemical state: intravital multiphoton fluorescence lifetime imaging of inflamed human skin,” Sci. Rep. 6, 22789 (2016). SRCEC3 2045-2322 10.1038/srep22789 27004454 PMC4804294

[25] Pastore M. N. et al., “Non-invasive metabolic imaging of melanoma progression,” Exp. Dermatol. 26, 607–614 (2017). 10.1111/exd.2017.26.issue-7 27992081

[26] Yew E. Rowlands C. So P. T. C., “Application of multiphoton microscopy in dermatological studies: a mini-review,” J. Innovative Opt. Health Sci. 7, 1330010 (2014). 10.1142/S1793545813300103 PMC4112132 25075226

[27] Lukina M. M. et al., “*In vivo* metabolic imaging of mouse tumor models in response to chemotherapy,” Proc. SPIE 10069, 100692G (2017). PSISDG 0277-786X 10.1117/12.2252123

[28] Jones J. D. et al., “*In vivo* multiphoton microscopy detects longitudinal metabolic changes associated with delayed skin wound healing,” Commun. Biol. 1, 198 (2018). 10.1038/s42003-018-0206-4 30480099 PMC6242983

[29] Hayden C. G. J. et al., “Sunscreen penetration of human skin and related keratinocyte toxicity after topical application,” Skin Pharmacol. Physiol. 18, 170–174 (2005). 10.1159/000085861 15908756

[r30] Holmes A. M. et al., “Relative penetration of zinc oxide and zinc ions into human skin after application of different zinc oxide formulations,” ACS Nano 10, 1810–1819 (2016). ANCAC3 1936-0851 10.1021/acsnano.5b04148 26741484

[r31] Liu D. C. et al., “The human stratum corneum prevents small gold nanoparticle penetration and their potential toxic metabolic consequences,” J. Nanomater. 2012, 1–8 (2012).1687-4110 10.1155/2012/721706

[32] Breunig H. G. Weinigel M. König K., “*In vivo* imaging of ZnO nanoparticles from sunscreen on human skin with a mobile multiphoton tomograph,” BioNanoScience 5, 42–47 (2015). 10.1007/s12668-014-0155-4

[33] Haridass I. N. et al., “Cellular metabolism and pore lifetime of human skin following microprojection array mediation,” J. Controlled Release 306, 59–68 (2019). JCREEC 0168-3659 10.1016/j.jconrel.2019.05.024 31121279

[r34] Mohammed Y. H. et al., “Microneedle enhanced delivery of cosmeceutically relevant peptides in human skin,” PLoS One 9, e101956 (2014). POLNCL 1932-6203 10.1371/journal.pone.0101956 25033398 PMC4102460

[35] Wei J. C. J. et al., “Space- and time-resolved investigation on diffusion kinetics of human skin following macromolecule delivery by microneedle arrays,” Sci. Rep. 8, 17759 (2018). SRCEC3 2045-2322 10.1038/s41598-018-36009-8 30531828 PMC6288161

[36] Mohammed Y. H. et al., “Efficacy, Safety and Targets in Topical and Transdermal Active and Excipient Delivery,” Chapter 23 in Percutaneous Penetration Enhancers Drug Penetration into/through the Skin: Methodology and General Considerations, Dragicevic N. Maibach H. I., Eds., pp. 369–391, Springer, Berlin, Heidelberg (2017).

[37] Faunce T. et al., “Sunscreen safety: the precautionary principle, The Australian Therapeutic Goods Administration and nanoparticles in sunscreens,” NanoEthics 2, 231–240 (2008). 10.1007/s11569-008-0041-z

[38] Ryman-Rasmussen J. P. Riviere J. E. Monteiro-Riviere N. A., “Penetration of intact skin by quantum dots with diverse physicochemical properties,” Toxicol. Sci. 91, 159–165 (2006). 10.1093/toxsci/kfj122 16443688

[39] Mangelsdorf S. et al., “Comparative study of hair follicle morphology in eight mammalian species and humans,” Skin Res. Technol. 20, 147–154 (2014). 10.1111/srt.2014.20.issue-2 23800212

[40] Prow T. W. et al., “Quantum dot penetration into viable human skin,” Nanotoxicology 6, 173–185 (2012). 10.3109/17435390.2011.569092 21456897

[41] Warren S. C. et al., “Removing physiological motion from intravital and clinical functional imaging data,” Elife 7, e35800 (2018). 10.7554/eLife.35800 29985127 PMC6037484

[42] Prow T. W. et al., “Nanoparticles and microparticles for skin drug delivery,” Adv. Drug Delivery Rev. 63, 470–491 (2011). ADDREP 0169-409X 10.1016/j.addr.2011.01.012 21315122

[43] Labouta H. I. et al., “Gold nanoparticle penetration and reduced metabolism in human skin by toluene,” Pharm. Res. 28, 2931–2944 (2011). 10.1007/s11095-011-0561-z 21833791

[44] Holmes A. M. et al., “Varying the morphology of silver nanoparticles results in differential toxicity against micro-organisms, HaCaT keratinocytes and affects skin deposition,” Nanotoxicology 10, 1503–1514 (2016). 10.1080/17435390.2016.1236993 27636544

[45] Song Z. et al., “Background free imaging of upconversion nanoparticle distribution in human skin,” J. Biomed. Opt. 18(6), 061215 (2013). JBOPFO 1083-3668 10.1117/1.JBO.18.6.061215 23183656

[46] Roberts M. S. et al., “*In vitro* and *in vivo* imaging of xenobiotic transport in human skin and in the rat liver,” J. Biophotonics 1, 478–493 (2008). 10.1002/jbio.200810058 19343674

[47] Becker & Hick “Application Note: Combined Fluorescence and Phosphorescence Lifetime Imaging (FLIM/PLIM) with the Zeiss LSM 710 NLO Microscopes,” 2018, https://www.becker-hickl.com/wp-content/uploads/2018/12/plim-zeiss-lsm710-v01.pdf (accessed 24 11 2019).

[48] Chelushkin P. S. Tunik S. P., “Phosphorescence lifetime imaging (PLIM): State of the art and perspectives,” in Progress in Photon Science: Recent Advances, Yamanouchi K. Tunik S. Makarov V., Eds., pp. 109–128, Springer International Publishing, Cham, Switzerland (2019).

[49] Friends of the Earth, “Nanotechnology and sunscreens: a consumer guide for avoiding nano-sunscreens,” 2007, https://foe.org/resources/nanotechnology-sunscreens-a-consumer-guide-for-avoiding-nanosunscreens/ (accessed 13 9 2019).

[50] Lademann J. et al., “Optical investigations to avoid the disturbing influences of furrows and wrinkles quantifying penetration of drugs and cosmetics into the skin by tape stripping,” J. Biomed. Opt. 10(5), 054015 (2005). 10.1117/1.2055507 16292975

[51] Raphael A. P. et al., “Zinc oxide nanoparticle removal from wounded human skin,” Nanomedicine 8, 1751–1761 (2013).1743-5889 10.2217/nnm.12.196 23463920

[52] Tinkle S. S. et al., “Skin as a route of exposure and sensitization in chronic beryllium disease,” Environ. Health Perspect. 111, 1202–1208 (2003). EVHPAZ 0091-6765 10.1289/ehp.5999 12842774 PMC1241575

[53] Gulson B. et al., “Small amounts of zinc from zinc oxide particles in sunscreens applied outdoors are absorbed through human skin,” Toxicol. Sci. 118, 140–149 (2010). 10.1093/toxsci/kfq243 20705894

[54] Gulson B. et al., “Comparison of dermal absorption of zinc from different sunscreen formulations and differing UV exposure based on stable isotope tracing,” Sci. Total Environ. 420, 313–318 (2012). 10.1016/j.scitotenv.2011.12.046 22316633

[55] Sharma V. et al., “Zinc oxide nanoparticle induced genotoxicity in primary human epidermal keratinocytes,” J. Nanosci. Nanotechnol. 11, 3782–3788 (2011). JNNOAR 1533-4880 10.1166/jnn.2011.4250 21780369

[r56] Sharma V. Anderson D. Dhawan A., “Zinc oxide nanoparticles induce oxidative DNA damage and ROS-triggered mitochondria mediated apoptosis in human liver cells (HepG2),” Apoptosis 17, 852–870 (2012). 10.1007/s10495-012-0705-6 22395444

[57] Akhtar M. J. et al., “Zinc oxide nanoparticles selectively induce apoptosis in human cancer cells through reactive oxygen species,” Int. J. Nanomed. 7, 845–857 (2012). 10.2147/IJN.S29129 PMC3289443 22393286

[58] Ghukasyan V. V. Kao F.-J., “Monitoring cellular metabolism with fluorescence lifetime of reduced nicotinamide adenine dinucleotide,” J. Phys. Chem. C 113, 11532–11540 (2009). JPCCCK 1932-7447 10.1021/jp810931u

[r59] Sanchez W. Y. et al., “Analysis of the metabolic deterioration of *ex vivo* skin from ischemic necrosis through the imaging of intracellular NAD(P)H by multiphoton tomography and fluorescence lifetime imaging microscopy,” J. Biomed. Opt. 15(4), 046008 (2010). JBOPFO 1083-3668 10.1117/1.3466580 20799810

[60] Wang H. W. et al., “Differentiation of apoptosis from necrosis by dynamic changes of reduced nicotinamide adenine dinucleotide fluorescence lifetime in live cells,” J. Biomed. Opt. 13(5), 054011 (2008). JBOPFO 1083-3668 10.1117/1.2975831 19021391

[61] Matta M. K. et al., “Effect of sunscreen application under maximal use conditions on plasma concentration of sunscreen active ingredients: a randomized clinical trial,” JAMA 321, 2082–2091 (2019). JAMAAP 0098-7484 10.1001/jama.2019.5586 31058986 PMC6549296

